# Supplementary material for: Radiomics for Dynamic Lung Cancer Risk Prediction in USPSTF-Ineligible Patients
Source: Cancers (Basel). 2025 Oct 23;17(21):3406. doi: 10.3390/cancers17213406 (PMC12611041; doi:10.3390/cancers17213406)
Supplement: Supplementary file 1 [file cancers-17-03406-s001.zip › cancers-3900051-supplementary.pdf]

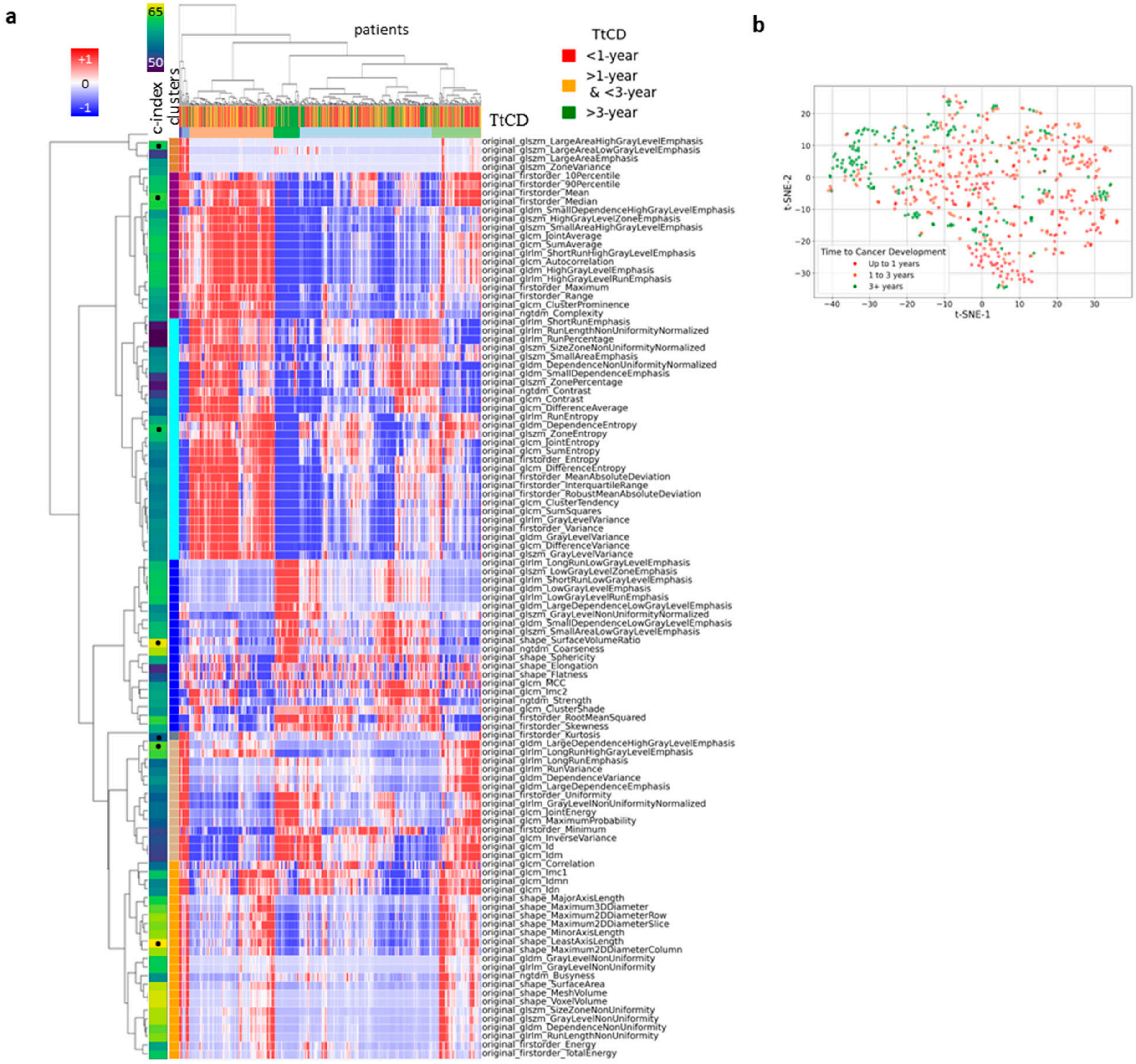

Figure S1. Feature selection procedure and t-SNE plot. (a) The hierarchical clustering-based feature selection process for radiomic features. Seven clusters are identified in this example, with indicating the selected features having the highest C-index in each cluster marked with a dot on the colormap. The colormap for TtCD (time to cancer development) represents the clustered columns, corresponding to patient samples. b) The t-SNE plot repre-sents features from the three distinct groups of patients. TtCD is shown for three distinct groups of patients: those who developed lung cancer within one year ("<1-year"), those who developed it between one and three years ("1-3 years"), and those whose diagnosis occurred more than three years after observation began (">3-year").

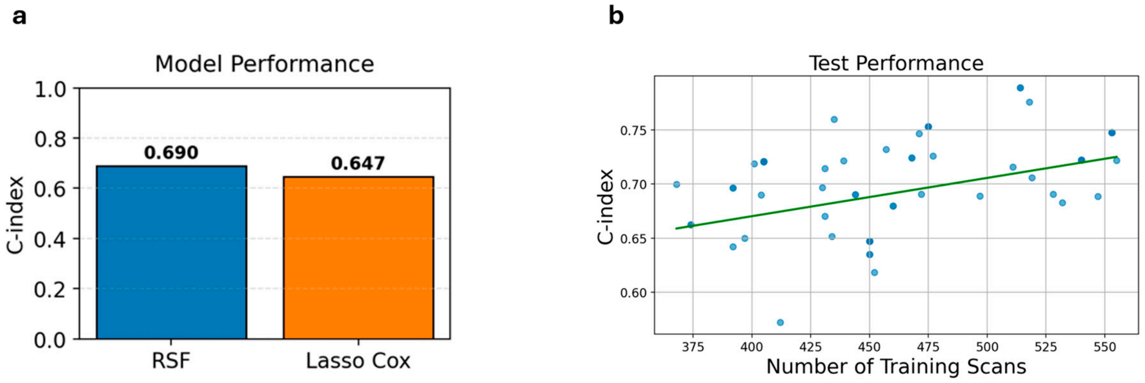

Figure S2. *(a)* Comparison of model performance between Random Survival Forest (RSF) and Lasso-Cox models across 100 nested cross-validation iterations, showing higher concordance for RSF (C-index = 0.690) compared with Lasso-Cox (C-index = 0.647). *(b)* Relationship between the number of training CT scans and model test performance, demonstrating a positive correlation between training sample size and C-index, indicating improved model stability and generalizability with increased data.

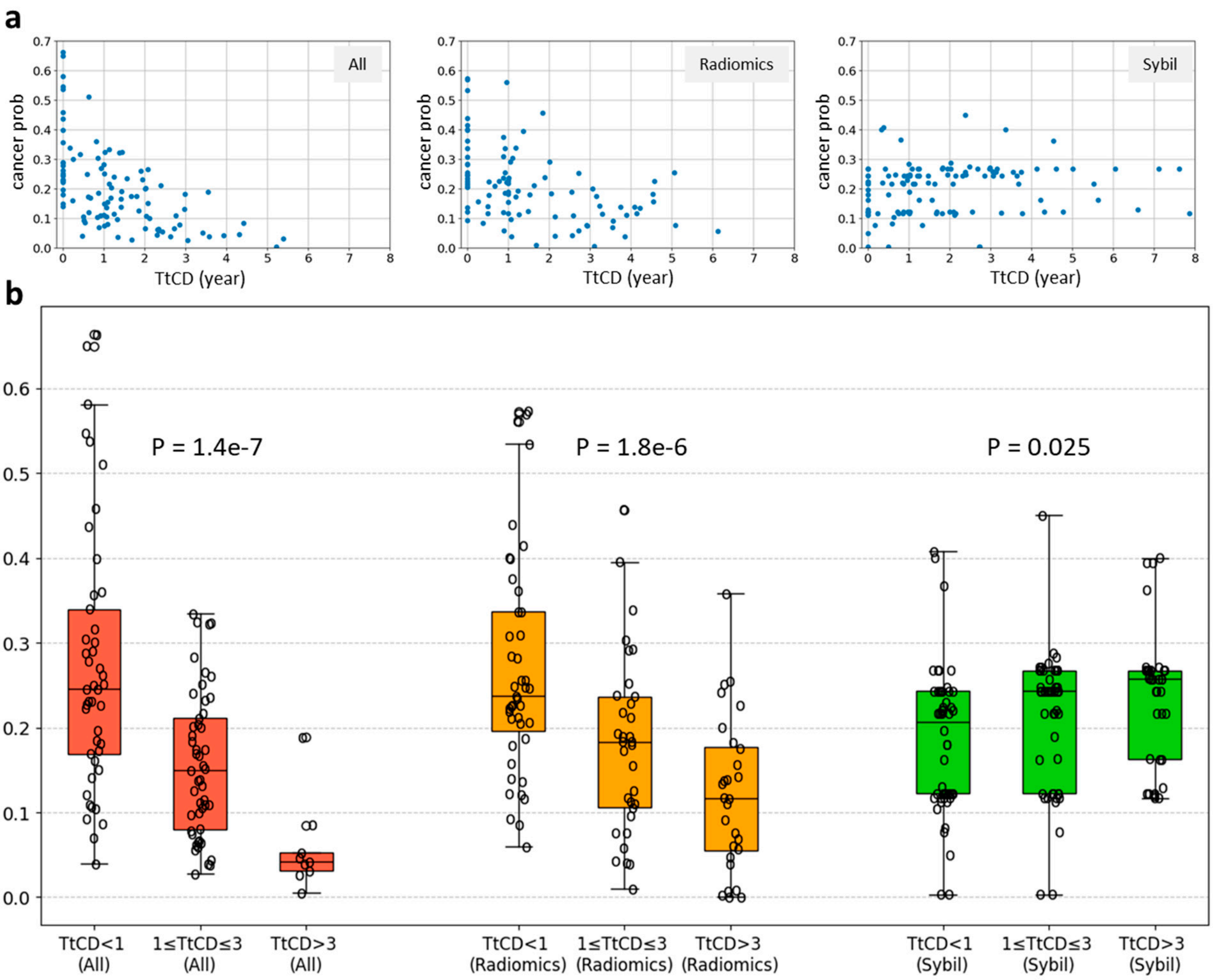

Figure S3. Comparison of predicted cancer development probability for different models on the test set. (a) Scatter plots showing the relationship between cancer development probability and true TtCD for three representative models from Figure 5b. (b) Corresponding box plots dividing the data into three-time intervals. The models were chosen based on median C-index performance in nested CV, leading to differences in test sets.
